# Assessing the Effect of a Food Voucher on the Dietary Intake of Patients with Diabetes Using the Canadian Diet History Questionnaire III: A Randomized Control Trial

**DOI:** 10.3390/nu17172865

**Published:** 2025-09-04

**Authors:** Adelaide Buadu, Moizza Zia Ul Haq, Lavanya Sinha, Areesha Sabir, Enza Gucciardi, Nav Persaud

**Affiliations:** 1 MAP Centre for Urban Health Solutions, Li Ka Shing Knowledge Institute, St. Michael’s Hospital, Unity Health Toronto, 209 Victoria St, Toronto, ON M5B 1T8, Canada; adelaide.buadu@unityhealth.to (A.B.); moizzazia.ulhaq@unityhealth.to (M.Z.U.H.); lavanya.sinha@unityhealth.to (L.S.);; 2 School of Nutrition, Toronto Metropolitan University, 350 Victoria Street, Toronto, ON M5B 2K3, Canada; egucciar@torontomu.ca; 3 Department of Family and Community Medicine, University of Toronto, 500 University Ave, Toronto, ON M5G 1V7, Canada

**Keywords:** CDHQ, food insecurity, type 2 diabetes mellitus, food prescription, healthy eating food index, diet quality, grocery food voucher

## Abstract

Background/Objectives: The high cost of healthy foods makes it difficult for people with a low income to manage diabetes. This study examined the effects of a monthly grocery voucher on the dietary intake, assessed through the Canadian Diet History Questionnaire III, of diabetes patients facing food or financial insecurity. We also assessed the impact on levels of hemoglobin A1c, beta-carotene, and ascorbic acid. Methods: Participants were randomly selected from a larger clinical trial and completed the survey at 6-month follow-up. Results: Voucher recipients consumed more whole fruit (mean difference in daily servings, MD 0.8; 95% CI [0.1, 1.6]) and fewer refined grains (MD −1.0; 95% CI [−1.9, −0.1]). For other food groups, the confidence intervals for the difference included null effect. Mean HEFI-2019 score was 51.7 out of 80, with voucher recipients averaging 52.4 vs. 51.0 for controls (MD 1.4; 95% CI [−3.6, 6.1]). The voucher group showed a slight HbA1c decrease (MD −0.4; 95% CI [−1.4, 0.5]). Conclusions: A voucher providing access to healthy foods for people with diabetes or prediabetes slightly increased intake of fruits and decreased intake of refined grains. Larger interventional studies are needed to determine the effects of vouchers on dietary intake among this population.

## 1. Introduction

The higher cost of foods that improve diabetes control is a barrier for people with a low income, and this may partially explain disparities in diabetes outcomes [1,2,3]. The relationship between nutrition and diabetes is well-established, with evidence indicating that dietary intake plays a crucial role in diabetes management [4,5]. Affordability is an important barrier to healthy eating, particularly among lower-income groups [6]. Healthier diets are generally associated with higher costs, while foods of lower nutritional value tend to be cheaper on a per-calorie basis [6,7]. This limits access among low-income populations and contributes to socioeconomic disparities in diet quality and health outcomes, such as type 2 diabetes [7]. One study noted that the price of healthy food was directly associated with insulin resistance and type 2 diabetes prevalence [8]. Low-income residents often substituted cheaper, energy-dense foods when healthier alternatives were cost-prohibitive [8]. Cost has been identified as a systemic barrier to healthy eating in low-income individuals with type 2 diabetes, and stress, affordability, and food preference have been found to lead to frequent consumption of unhealthy, low-cost options [9].

Vouchers providing free access to healthy foods are one of several interventions aimed at improving the health of individuals with diabetes facing food insecurity [10,11,12,13,14,15,16,17,18]. The results from trials of interventions that improve healthy food access, focusing on diabetes patients, suggest a benefit. For example, participants with improved access to healthy foods exhibited significant increases in dark green vegetable consumption [13] and overall fruit and vegetable intake compared to the control group [14]. These improvements were reflected in assessments such as the Starting the Conversation Diet evaluation [11] and the Healthy Eating Index [12]. Some trials involving healthy food vouchers demonstrated moderate increases in fruit and vegetable consumption post-intervention, though these changes were not statistically significant. One study found significant increases in green vegetable consumption, but changes in orange vegetable and fruit intake did not reach significance [15]. In a study focused on low-income groups, fruit and vegetable consumption significantly increased after three months of receiving vouchers, regardless of whether dietary advice was provided, emphasizing the value of combining financial support with nutrition guidance, especially for this socioeconomic group [16]. Research also suggests that food prescription programs can effectively lower diabetes markers in individuals with type 2 diabetes. Some trials have reported improvements in hemoglobin A1c (HbA1c) levels following increased access to healthy foods [11,17], while others have shown varied results [13,14]. Notably, one study found that participants with higher baseline HbA1c levels experienced significant reductions after a six-month food prescription intervention [18]. A clinical trial of the effects of healthy food vouchers found improved diabetes control [17].

The purpose of this substudy of a clinical trial was to determine the effect of a $65 monthly voucher on the dietary intake, using the Canadian Diet History Questionnaire, of patients with diabetes who experience food or financial insecurity. The larger trial found that the voucher was associated with a larger reduction in HbA1c than usual care, but the difference was not statistically significant. To our knowledge, no existing research has specifically investigated the effects of food vouchers on dietary intake among individuals with diabetes, using the Canadian Diet History Questionnaire (CDHQ) III. The CDHQ offers significant advantages for food analysis, especially when estimating average daily dietary intake over extended periods, such as one month [19]. We not only assess dietary adherence through serving counts but also incorporate a plate-model approach, as suggested by the Canadian Food Guidelines (e.g., HEFI-2019).

## 2. Materials and Methods

Participants were randomly selected from a larger clinical trial focused on diabetes control as measured through serum HbA1c values, which is reported separately. This randomized controlled trial included primary care patients, aged 19 years and older, who had either diabetes or prediabetes and reported experiencing food insecurity or difficulty making ends meet. The trial was registered on 16 March 2023 (ClinicalTrials.gov: NCT05776420). Participants who provided consent at baseline to be contacted for the CDHQ study were randomly selected to complete the CDHQ survey at the 6-month follow-up. Surveys were conducted between September 2023 and November 2024. 

Intervention participants were provided with a reloadable voucher in the form of a supermarket gift card. The voucher was reloaded by $65 (or $85 for participants who lived in a household of 6 or more individuals) each month for 6 months. The vouchers could be used to purchase anything sold at the supermarket, but study personnel instructed the participants to only use the voucher for purchasing fruits and vegetables. We describe the intervention in more detail elsewhere [20].

The CDHQ III represents an adaptation of the DHQ III, specifically modified to better align with Canadian dietary patterns. This comprehensive questionnaire asks participants to detail their dietary intake over the past month, including what they consumed, the quantities, and the frequency of consumption [19]. The collected responses are used to calculate caloric intake, nutrient levels, and food group consumption. Unlike earlier versions of the DHQ and CDHQ, which necessitated the use of DietCalc analysis software, the analysis for both the DHQ III and CDHQ III has been seamlessly integrated into a web-based platform. This advancement allows for the automatic calculation of daily nutrient and food group intakes, which can be accessed through the Researcher website. It is important to highlight that while nutrient data is automatically available for CDHQ III, additional steps are required to derive food group intakes. The grams and percentage of calories (% kcal) for various nutritional components were calculated, including energy (calories), total fat, saturated fat, unsaturated fat, protein, carbohydrates, and sodium. The total daily food consumed (amount in grams) was also assessed. The CDHQ III output file was extracted for further analysis of the food group consumption.

Several data sources have been utilized to facilitate the calculation of food group intakes. These sources comprise a comprehensive analysis file that includes nutrient and food intake information for every participant (available on the CDHQ III Researcher website), the CDHQ III nutrient database along with its related files, the Health Canada report titled “Classification of Foods in the Canadian Nutrient File According to Eating Well with Canada’s Food Guide” [21], and the detailed food and recipe files obtained from the Canadian Community Health Survey (CCHS) 2015. Reference was also made to the study conducted by McInerney and colleagues regarding the incorporation of food group equivalents in the CDHQ II for estimating the Canadian Healthy Eating Index-2005 [22].

The 296 food categories in the CDHQ III nutrient database were organized into four distinct groups: (1) simple foods, (2) mixed/recipe foods, (3) fat-added foods, and (4) other foods. The simple foods category comprises primarily single food items (e.g., apples, yogurt, bread). The mixed/recipe foods category encompasses dishes containing multiple foods (e.g., pizza with meat sauce, sandwiches). The fat-added foods category represents simple items prepared with added fat (e.g., vegetables with added fat). The other foods category includes items that do not fall under the previous categories or are not typically consumed as standalone items (e.g., cream cheese, ketchup). Ten new variables representing food group equivalents (Canadian Food Guidelines servings) were created for each of the 296 food categories. By utilizing the Canadian Nutrient File (CNF)/Canadian Food Guidelines (CFG) classification and the accompanying database, the nutrient profile of each food was evaluated to determine the primary food, the weight of one standard CFG serving, and the appropriate CFG food group(s) to which the primary food should be assigned. To produce the output for the 10 CFG food groups (e.g., CFG servings for total fruits and vegetables, whole fruits, dark green and orange vegetables, white potatoes, milk and alternatives, meat and alternatives, total grain products, whole grains, and refined grains), the weight (in grams) of each CDHQ III portion size was divided by the weight (in grams) of 1 CFG serving of the identified primary food. In instances where the primary food could not be located within the CNF/CFG classification or when the specific type of food was unclear (for example, “cheese” without further description), the standard weight for that food type from the CFG was utilized (e.g., the CFG designates a serving weight of 50 g for cheese). Alternatively, a consultation among the authors helped identify the most suitable CFG food group allocations. When dealing with multiple primary foods, we used similar methods to those described in a study examining approaches for quantifying diet quality using the CDHQ [22]. For cases involving multiple primary foods, which was often the situation with mixed or recipe foods, we first evaluated the ingredients (e.g., type of vegetable, grain) based on research and the authors’ familiarity with typical ingredients in mixed food recipes. We made informed assumptions regarding the main components and their corresponding food groups. For instance, in the case of a dish like poutine, we identified potato fries as the primary ingredient contributing to the vegetables and fruits CFG group. Subsequently, we calculated the total gram weight of each ingredient in the mixed food using the weight (g) from the CDHQ III portion size, before dividing by the standard CFG serving weight based on the identified primary food(s) and their respective CFG food group(s).

The Healthy Eating Food Index (HEFI-2019) has undergone validation to assess compliance with the revised dietary guidelines for Canadians effectively [23], and as such, aided in the exploration of the research question at hand. To compute the HEFI-2019, the initial step involved categorizing the consumed foods and beverages into distinct groups utilized for determining both the numerator and denominator of each HEFI-2019 component. Subsequently, the intake quantities of food (expressed in recommended allowance (RA)) and beverages (measured in grams) for each category were established. Following this, total nutrient intakes (including mono- and polyunsaturated fats, saturated fats, free sugars in grams, sodium in milligrams) and total energy (measured in calories) were calculated [24]. This data was subsequently used to establish the final HEFI-2019 score. The algorithm for scoring and the SAS macros are accessible to the public (SAS software. Version 9.4. Cary, NC, USA: SAS Institute Inc., 2023) [25]. Detailed information on the HEFI-2019 components, the point allocation system, and the scoring thresholds is available in Table 1. Points that fell between the minimum and maximum scores were awarded proportionately across all components.

The HEFI-2019 comprises 10 components with an overall maximum score of 80 points. Each component is represented as ratios (for instance, proportions of total foods, total beverages, or total energy). The Vegetables and Fruits component was evaluated as the ratio of vegetables and fruits to total foods, specifically including whole, dried, and canned varieties, while excluding fruit juices, which are categorized as sugary drinks per CFG-2019. The benchmark of 50% vegetables and fruits was derived from the CFG-2019 snapshot, with a maximum of 20 points awarded when the ratio of vegetables and fruits in RAs to total foods in RAs is at least 0.5, indicating that half or more of food consumption consists of these items. The Whole-Grain Foods component was scored based on the ratio of whole-grain foods (the first ingredient must be a whole grain or whole wheat) to total foods, with a benchmark of 25% whole-grain foods from CFG-2019, granting a maximum of 5 points for ratios ≥ 0.25. The Grain Food Ratio was determined as the ratio of whole-grain foods to the aggregate of all grain foods (both measured in RAs), where a ratio of 1 signifies that all grain foods consumed are whole-grain, yielding the maximum of 5 points in alignment with CFG-2019 recommendations. The Protein Foods component was calculated as the ratio of protein foods (including fish, shellfish, eggs, poultry, red meat, dairy products like unsweetened milk, and plant-based protein foods, including unsweetened alternatives) to total foods. Sugar-sweetened milk and sugary plant-based beverages were excluded as they fall under sugary drinks, as defined by CFG-2019 [26]. A benchmark of 25% protein food representation from the CFG-2019 snapshot set the scoring standard; thus, ratios ≥ 0.25 received the maximum of 5 points. For the Plant-Based Protein component, the scoring was based on the ratio of plant-based protein foods to total protein foods, with a maximum of 5 points awarded for a ratio exceeding 0.50.

The Beverages component assessed the ratio of water (including carbonated forms) and other unsweetened beverages (like unsweetened tea or coffee, unsweetened milk, and unsweetened plant-based beverages) to total beverage intake, all converted to grams. The denominator includes all previously mentioned drinks in addition to sugary drinks, artificially sweetened beverages, and alcohol. The ideal target is 0 g per day of sugary drinks, artificial sweeteners, or alcohol, which ensures that a maximum score of 10 points is given when the ratio equals 1.0. The scoring for the Fatty Acids component was derived from the ratio of unsaturated to saturated fats, with ratios of ≥2.6 yielding a maximum of 5 points. The Saturated Fats component was calculated from the percentage of energy (%E) derived from saturated fats, awarding the maximum 5 points for values below 10%E, in accordance with CFG-2019 recommendations. Free sugars were also critically assessed as a nutrient of concern under CFG-2019, with intakes below 10%E receiving the full 10 points. Finally, the Sodium component employed a density-adjusted approach, awarding maximum points when the sodium-to-energy intake ratio is <0.9 (2300 mg/2600 kcal). Further detailed methodologies and considered factors are documented elsewhere [24,26].

### Statistical Analyses

We compared reported dietary intake between the intervention and control groups. The chi-square test and Fisher’s exact test for independence were utilized to assess significant differences between categorical variables. Descriptive statistics (mean, mean differences, standard deviations, minimums, and maximums) were computed for various variables, including food group equivalents (CFG servings) for total fruits and vegetables, whole fruit, dark green and orange vegetables, white potatoes, milk and alternatives, meat and alternatives, total grain products, whole grains, and refined-grain foods, as well as nutritional components such as energy (calories), total fat, saturated fat, unsaturated fat, protein, carbohydrates, and sodium, in addition to food amount, ultra-processed food intake, and HEFI-2019 scores. We analyzed outcome variables from the larger clinical trial, calculating the mean difference for A1c, beta-carotene, and ascorbic acid, along with the proportions of participants reporting fruit and vegetable intake, financial stability, food security, and overall health. The larger trial was powered to detect a 0.4% difference in A1c; no sample size estimation was performed for this substudy.

Student *t*-tests were applied to compare mean differences. Statistical significance was determined at *p* < 0.05 and a 95% confidence interval not enclosing “0” (value representing “no effect”). We do not correct *p*-values for multiple comparisons. Analyses were performed using Stata (StataCorp. 2019. Stata Statistical Software: Release 16.1, StataCorp LLC., College Station, TX, USA).

## 3. Results

This substudy included a total of 40 participants with diabetes who were experiencing food insecurity: 20 who received a voucher for healthy foods (7 males and 13 females) and 20 control participants (10 males and 10 females), with a mean age of 57 years. Most participants had diabetes (72.5%, *n* = 29) and the remainder (27.5%, *n* = 11) had prediabetes. Most (60%, *n* = 24) participants had an annual income of less than $30,000, with 14 in the control group and 10 in the intervention group (see Table 2).

Overall, no substantial differences were observed between the two trial arms for any of the nutritional components, including energy (calories), total fat, saturated fat, unsaturated fat, protein, carbohydrates, and sodium (see Table 3). The intake of whole fruit was higher among voucher recipients compared to control participants (mean [standard deviation (SD)] 2.2 [1.4] and 1.3 [1.05]; mean difference (MD): 0.8, 95% CI [0.1, 1.6]). Participants who received vouchers reported consuming slightly more daily servings of total vegetables and fruits compared to the control group, but the confidence intervals for the difference were wide and included no difference (6.4 [3.9] and 6.2 [5.6]; MD: 0.2, 95% CI [−2.9, 3.3]). The mean daily servings of whole grains were higher among voucher recipients, although the confidence intervals for the difference included no effect (0.7 [0.8] and 0.4 [0.5]; MD: 0.3, 95% CI [−0.2, 0.7]). Participants receiving the voucher consumed fewer refined grains than those in the control arm (1.2 [1.0] vs. 2.2 [1.8]; MD: −1.0, 95% CI [−1.9, −0.1]). For various CFG food categories (including dark green vegetables, orange vegetables, white potatoes, dairy, and meat alternatives), average daily servings were similar between the two arms. In terms of ultra-processed food consumption, those receiving the vouchers reported lower intakes compared to their control participants, but the confidence intervals for the difference were wide and included no effect (59.6 [51.2] and 73.3 [76.0]; MD: −13.7, 95% CI [−55.1, 27.8]). Sex differences are presented in Table S1.

The mean HEFI-2019 score, measuring overall diet quality, was 51.7 (SD 7.3, 95% CI [49.3, 54.0]). The HEFI-2019 score was slightly higher for voucher recipients (52.4 [6.7]) than for control participants (51.0 [8.1]), but the confidence intervals for the difference were wide and included no effect (MD: 1.4; 95% CI [−3.4, 6.1]). Individuals receiving vouchers had higher intake scores for vegetables and fruits, 18.2 (3.3) points, compared to controls, 16.2 (4.1) points (out of 20), though the confidence intervals for the difference included no effect (MD: 2.0; 95% CI [−0.4, 4.4]). In terms of the HEFI-2019 components, participants in the intervention group had higher scores than control participants in 7 out of 10 components, but the confidence intervals for the differences were wide and included no effect (see Table 4). Differences between males and females are presented in Table S2. Overall, for our sample of 40 participants, CFG food groups and HEFI-2019 components revealed mean daily intakes that fell below the recommended levels set forth by the CFG guidelines.

At baseline, the intervention group in this substudy of a larger clinical trial had a higher A1c. During the 6-month follow-up period, A1c slightly decreased in the group receiving the voucher, but slightly increased in the control group, and the between-group difference had wide confidence intervals and lacked statistical significance (change in A1c mean difference (MD) −0.4; 95% CI [−1.4, 0.5]; *p* = 0.34; see Table 5). The differences in serum beta-carotene and ascorbic acid concentrations were not substantial, with beta-carotene showing an MD of −0.1 (95% CI [−0.3, 0.1]) and ascorbic acid an MD of 3.1 (95% CI [−10.3, 16.5]). No substantial differences were noted in vegetable consumption between the voucher recipients and controls (both reported consuming vegetables two or more times per day at a rate of 75%). Voucher recipients reported a higher frequency of fruit consumption at follow-up compared to control participants (45% vs. 25% consuming two or more servings daily), but this difference was not significant. There were no differences in self-reported food insecurity, financial insecurity, or general health between voucher recipients and controls. Among the 20 intervention participants, 14 used all of the voucher, and only one participant used less than 50% of the total voucher value.

## 4. Discussion

This substudy of a six-month clinical trial found that participants with type 2 diabetes or prediabetes who received small monthly grocery vouchers displayed a slight increase in daily servings of fruits and vegetables, but there was substantial uncertainty in the estimate of the difference and the clinical importance of the slight difference is unclear. Those allocated to receive the vouchers reported greater whole fruit consumption and less refined grain intake. There were no substantial differences in overall or whole grain consumption. The overall diet quality, as assessed by the HEFI-2019 index score, was slightly higher among voucher recipients, although this difference was not substantial. We did not observe differences between study groups in most other food group categories and nutritional components. Objective measures, including HbA1c, showed no difference, and there was considerable variability in biomarkers for vegetable and fruit consumption within this small substudy.

Other randomized control trials in similar populations of interventions that improved access to healthy foods found significant improvements in mean daily overall fruit and vegetable consumption [14], increased fruit consumption [12,13,29], or increased vegetable consumption [12] among intervention participants compared to controls. These findings align with our main randomized control trial, where intervention participants had increased self-reported vegetable consumption and self-reported fruit consumption compared to controls [20]. Our CDHQ data indicated an increase in whole fruit intake among the subgroup of 20 voucher recipients. Notably, only one completed randomized controlled trial measured the effect of monetary support in the form of vouchers; other trials report the effect of other interventions that directly improve food access through medically tailored meal deliveries, food-as-medicine programs, or food boxes. The effects of different intervention types may vary.

Trials that compared overall diet quality using survey-based index scores, the Starting the Conversation Diet assessment, and the Healthy Eating Index, reported improved overall index scores among intervention participants compared to controls [11,13,14]. Furthermore, a trial examining the impacts of medically tailored meals on Healthy Eating Index scores reported higher intake of whole fruit among intervention participants compared to controls, which aligns with our findings [12]. Higher intakes of whole grain, dark green vegetables, protein, and fatty acids, as well as lower sodium intake amongst intervention participants, have been reported in other trials; however, we did not find substantial differences in these measures between intervention and control participants [12]. Variations in the findings of the current study compared to previous trials may stem from the methodological differences in food provision strategies. Most of these trials examined the impacts of direct healthy food provision, through medically tailored meal delivery or provision of healthy food boxes at clinics of food banks or pantries, on dietary intake. Outcomes of voucher-based interventions may differ from those of interventions that directly provide healthy foods. With vouchers, study participants are required to make their own decisions regarding which foods are best suited to their dietary and health needs. Additionally, a duration of 6 months may be insufficient to capture significant changes in dietary habits.

The statistical power of this study is limited due to a small sample size, and the multiple statistical comparisons performed mean that some observed differences may be due to chance. This study is subject to limitations typical of self-reported dietary assessments, and it was also an open-label clinical trial where participants were aware of their group allocation. Common challenges associated with dietary surveys that rely on self-reporting, such as potential biases of under- or over-reporting [30,31], are expected with CDHQ III. This misreporting can affect the accuracy of servings based on Canada’s Food Guide (CFG) and the Healthy Eating Food Index-2019 (HEFI-2019), potentially leading to both underestimation and overestimation of specific food group servings. The actual value of the voucher diminished after the trial was designed and funded, due to rising food costs [32]. As a result, the voucher amount may not have been sufficient to effect meaningful changes in diet, which could also explain the lack of significant differences in food group intakes between the two groups. Participants in the same group might have received supports similar to the voucher and as such, contamination might have reduced the difference between groups. Furthermore, seasonal variations in food prices and availability may influence both dietary intake and biomarker outcomes. We did not include all measures of potential interest such as weight, blood pressure, and cholesterol levels. We also did not assess food literacy or nutritional knowledge. Longer-term effects cannot be determined from this trial which followed participants for 6 months.

## 5. Conclusions

Grocery vouchers may slightly help individuals with prediabetes or diabetes to align their diets with established nutritional guidelines, especially with regard to fruit and the consumption of refined grains. Interventions designed to promote access to healthier food options may help to address inequities in health outcomes experienced by those with diabetes. Larger interventional studies will help better characterize the effects of these interventions across diverse populations.

## Figures and Tables

**Table 1:** HEFI-2019 components, points allocated and the standards for scoring.

	Component Name	Measurement (Ratio)	Maximum Points	Unit	Standard Minimum Score	Standard Maximum Score
1	Vegetables and fruits	Total vegetables and fruits ^1^/total foods ^2^	20	RA/RA	No vegetables and no fruits	≥0.50
2	Whole-grain foods	Total whole-grain foods ^3^/total foods ^2^	5	RA/RA	No whole-grain foods	≥0.25
3	Grain foods ratio	Total whole-grain foods ^3^/total foods ^4^	5	RA/RA	No whole-grain foods	1.0
4	Protein foods	Total protein foods ^5^/total foods ^2^	5	RA/RA	No protein foods	≥0.25
5	Plant-based protein foods	Plant-based protein foods ^6^/total protein foods ^5^	5	RA/RA	No plant-based foods	≥0.50
6	Beverages	(Plain water including carbonated + unsweetened beverages) ^7^/total beverages ^8^	10	g/g	No water and no unsweetened beverages	1.0
7	Fatty acids ratio	(Mono- + polyunsaturated fat)/total saturated fat	5	g/g	≤1.1	≥2.6
8	Saturated fats	Total saturated fat/energy	5	%E (kcal/kcal)	≥15%E	<10%E
9	Free sugars	Total free sugars/energy	10	%E (kcal/kcal)	≥20%E	<10%E
10	Sodium	Total sodium/energy	10	mg/kcal	≥2.0	<0.9

Note: Sourced from [24]. ^1^ All vegetables and fruits, excluding fruit juice. ^2^ Includes all foods consumed plus beverages considered in protein foods (i.e., unsweetened milk and unsweetened plant-based beverages that contain protein); excludes all other beverages, solid fats, oils and spreads, and culinary ingredients (e.g., spices). ^3^ Foods where the first ingredient is either whole grain or whole wheat. ^4^ All grain foods. ^5^ All protein foods, excluding sweetened milk. ^6^ All plant-based protein foods. ^7^ All unsweetened beverages (e.g., water, tea, coffee, milk, and plant-based beverages). ^8^ Sum of all types of beverages, including alcoholic drinks. RA, recommended allowance; %E, percentage of energy.

**Table 2:** Demographic Characteristics of the Substudy Participants.

	Total *n* = 40	Male *n* = 17	Female *n* = 23	Control *n* = 20	Intervention *n* = 20
Age, mean yrs (SD)	57.3 (13.8)	55.3 (10.5)	58.8 (15.9)	57.3 (14.1)	57.3 (13.8)
*p*-value		0.44 *		1.00 *	
Sex, *n* (%)					
Male	17 (42.5)	-	-	10 (50.0)	7 (35.0)
Female	23 (57.5)	-	-	10 (50.0)	13 (65.0)
*p*-value				0.34 ^a^	
Condition, *n* (%)					
Pre-diabetes	11 (27.5)	4 (23.5)	7 (30.4)	7 (35.0)	4 (20.0)
Diabetes	29 (72.5)	13 (76.5)	16 (69.6)	13 (65.0)	16 (80.0)
*p*-value		0.73		0.48	
Income Level, *n* (%)					
<$30,000	24 (60.0)	10 (58.8)	14 (60.9)	14 (70.0)	10 (50.0)
$30,000–$79,999	13 (32.5)	6 (35.3)	7 (30.4)	5 (25.0)	8 (40.0)
$80,000+	3 (7.5)	1 (5.9)	2 (8.7)	1 (5.0)	2 (10.0)
*p*-value		1.00		0.47	

* *p*-values for continuous variables were evaluated using two-sample *t*-test; *p*-values for categorical variables were evaluated via chi-square ^a^ and Fisher’s exact test. A *p*-value < 0.05 was considered statistically significant.

**Table 3:** Distribution of daily intake of food group equivalents (in Canada’s Food Guide servings) and nutrients according to trial groups, as reported in the Canadian Diet History Questionnaire (CDHQ) III.

	Control		Intervention			
Total Sample	Mean (SD)	Min, Max	Mean (SD)	Min, Max	Mean Differences (95% CI) *	*p*-Values *
CFG Equivalent in Servings ^1^						
Total fruit and vegetables ^2,3^	6.2 (5.6)	1.0, 20.5	6.4 (3.9)	1.5, 15.3	0.2 (−2.9, 3.3)	0.90
Whole fruits ^4^	1.3 (1.1)	0.2, 3.8	2.2 (1.4)	0.5, 5.4	0.8 (0.1, 1.6)	0.04
Dark green vegetables ^2^	1.1 (1.6)	0.0, 6.6	1.1 (1.0)	0.0, 2.9	0.0 (−0.9, 0.9)	0.98
Orange vegetables ^2^	0.4 (0.5)	0.0, 1.9	0.3 (0.3)	0.0, 1.2	0.0 (−0.3, 0.2)	0.68
White potato vegetables ^5^	0.3 (0.4)	0.0, 1.8	0.3 (0.2)	0.0, 0.8	0.0 (−0.2, 0.2)	0.85
Total grains ^2^	3.2 (2.0)	0.7, 7.8	2.6 (2.0)	0.3, 7.2	−0.6 (−1.9, 0.7)	0.36
Whole grains ^6^	0.4 (0.5)	0.0, 2.1	0.7 (0.8)	0.0, 3.5	0.3 (−0.2, 0.7)	0.22
Refined grains ^7^	2.2 (1.8)	0.4, 7.1	1.2 (1.0)	0.2, 3.6	−1.0 (−1.9, −0.1)	0.04
Milk and alternatives ^2,8^	1.3 (1.1)	0.2, 3.9	0.8 (0.6)	0.1, 2.2	−0.4 (−1.0, 0.1)	0.11
Meat and alternatives ^2,9^	2.6 (2.0)	0.5, 6.7	2.1 (1.5)	0.3, 4.7	−0.5 (−1.6, 0.6)	0.36
Other Foods and Nutrients						
Ultra-processed foods in grams ^10^	73.3 (76.0)	14.5, 323.0	59.6 (51.2)	13.2, 217.9	−13.7 (−55.1, 27.8)	0.51
Amount of food in grams	3138.8 (2620.9)	681.2, 12,696.1	3704.7 (2761.3)	651.2, 11,349.8	565.9 (−1157.4, 2289.3)	0.51
Energy in kcal	1307.3 (706.5)	380.4, 2753.8	1164.2 (513.9)	246.0, 1916.5	−143.1 (−538.5, 252.4)	0.47
Total fat in grams	48.6 (32.0)	13.2, 134.6	43.8 (24.2)	7.0, 84.8	−4.9 (−23.0, 13.3)	0.59
Total fat in % kcal	32.7 (6.8)	18.5, 45.7	32.5 (7.7)	14.4, 47.3	−0.2 (−4.9, 4.5)	0.94
Saturated fat in grams	15.0 (9.3)	4.7, 37.4	14.2 (9.4)	2.2, 34.2	−0.9 (−6.9, 5.1)	0.77
Saturated fat (% kcal)	10.3 (2.4)	6.0, 14.4	10.5 (3.8)	2.9, 16.7	0.2 (−1.9, 2.2)	0.85
Unsaturated fat in grams	33.6 (23.2)	8.5, 97.1	29.6 (15.4)	4.8, 57.5	−4.0 (−16.6, 8.6)	0.53
Unsaturated fat in % kcal	22.4 (5.2)	10.9, 31.7	22.1 (4.8)	11.5, 33.8	−0.4 (−3.6, 2.8)	0.82
Protein in grams	57.7 (35.4)	17.3, 123.2	48.7 (27.6)	9.5, 99.6	−8.9 (−29.2, 11.4)	0.38
Protein in % kcal	17.6 (4.6)	10.7, 33.6	16.1 (3.9)	7.4, 21.7	−1.6 (−4.3, 1.2)	0.26
Carbohydrates in grams	165.8 (90.7)	47.3, 385.5	149.6 (58.9)	38.4, 264.3	−16.2 (−65.2, 32.8)	0.51
Carbohydrates in % kcal	51.3 (10.0)	31.1, 69.4	53.8 (11.5)	37.4, 83.5	2.5 (−4.4, 9.4)	0.47
Sodium in mg	2128.7 (1527.0)	674.7, 6474.5	1837.8 (1088.9)	413.1, 4696.8	−290.9 (−1139.9, 558.0)	0.49

^1^ Servings according to [27]. ^2^ Foods acceptable in the food group according to CFG-2007. ^3^ Includes 100% fruit and vegetable juice. ^4^ Includes all forms except juice. ^5^ Regardless of cooking method (e.g., fries). ^6^ All grain foods reported as whole-grain in CDHQ III. ^7^ All grain foods not reported as whole-grain in CDHQ III. ^8^ Includes all milk products, such as fluid milk, yogurt, cheese, and fortified soy beverages. ^9^ Includes seafood, nuts, seeds, soy products (other than beverages), and legumes (beans and peas). ^10^ Ultra-processed foods as defined by [28]. * Statistically significant values were evaluated using a two-sample *t*-test at *p* < 0.05 and a 95% confidence interval not enclosing “0” (value representing “no effect”) for between-group differences in means (*n* = 40).

**Table 4:** Mean component scores on the Healthy Eating Food Index (HEFI)-2019 according to trial group, as reported in the Canadian Diet History Questionnaire (CDHQ) III.

	Control		Intervention			
Components (Maximum Points Possible)	Mean (SD)	Min, Max	Mean (SD)	Min, Max	Mean Differences (95% CI)	*p*-Values *
Food and Beverage Intake						
1 Vegetables and fruits (20)	16.2 (4.1)	5.9, 20.0	18.2 (3.3)	8.3, 20.0	2.0 (−0.4, 4.4)	0.09
2 Whole-grain foods (5)	0.8 (1.0)	0.0, 3.1	0.9 (0.9)	0.0, 2.6	0.1 (−0.5, 0.7)	0.74
3 Grain foods ratio (5)	1.0 (1.2)	0.0, 4.0	1.3 (1.2)	0.0, 3.9	0.3 (−0.5, 1.1)	0.46
4 Protein foods (5)	4.5 (1.2)	1.1, 5.0	4.2 (1.2)	1.4, 5.0	−0.3 (−1.1, 0.4)	0.38
5 Plant-based protein foods (5)	0.9 (1.2)	0.0, 4.8	1.1 (1.5)	0.0, 5.0	0.2 (−0.6, 1.1)	0.58
6 Beverages (10)	8.4 (1.8)	2.9, 10.0	8.4 (2.5)	1.3, 10.0	0.1 (−1.3, 1.4)	0.93
Subgroup 1 Total Score	31.7 (5.7)	19.4, 41.6	34.1 (4.7)	24.6, 41.01	2.4 (−1.0, 5.7)	0.16
Nutrient Intake						
7 Fatty acids ratio (5)	2.6 (1.4)	0.2, 5.0	2.9 (1.8)	0.2, 5.0	0.3 (−0.8, 1.3)	0.61
8 Saturated fats (5)	3.9 (1.6)	0.6, 5.0	3.2 (2.1)	0.0, 5.0	−0.6 (−1.8, 0.5)	0.28
9 Free sugars (10)	8.5 (2.2)	1.8, 10.0	7.6 (3.3)	0.0, 10.0	−0.9 (−2.7, 0.9)	0.29
10 Sodium (10)	4.2 (2.8)	0.0, 10.0	4.6 (3.2)	0.0, 9.9	0.3 (−1.6, 2.3)	0.73
Subgroup 2 Total Score	19.3 (3.9)	12.6, 24.8	18.3 (3.6)	10.4, 27.4	−1.0 (−3.4, 1.4)	0.40
HEFI Total Score (80)	51.0 (8.1)	35.0, 65.1	52.4 (6.7)	35.9, 65.5	1.4 (−3.4, 6.1)	0.56

* Statistically significant values were evaluated using a two-sample *t*-test at *p* < 0.05 and a 95% confidence interval not enclosing “0” (value representing “no effect”) for between-group differences in means (*n* = 40).

**Table 5:** A1c and other outcome results by group.

	Control *n* = 20	Intervention *n* = 20	Mean Difference (95% CI)	*p*-Values
A1c values, mean (SD)				
Baseline HbA1c	7.0 (1.0)	7.8 (1.7)	0.9 (0.0, 1.7)	0.05
6-month HbA1c	7.4 (1.6)	7.8 (1.4)	0.4 (−0.5, 1.4)	0.36
Change in HbA1c	0.4 (1.4)	0.0 (1.5)	−0.4 (−1.4, 0.5)	0.34
Self-reported vegetable consumption, *n* (%)				
Less than 2 times per day	15 (75)	15 (75)	-	
2 or more times per day	5 (25)	5 (25)	-	1.00
Self-reported fruit consumption, *n* (%)				
Less than 2 times per day	15 (75)	11 (55)	-	
2 or more times per day	5 (25)	9 (45)	-	0.18
Serum β-carotene, mean (SD)	0.4 (0.4)	0.3 (0.3)	−0.1 (−0.3, 0.1)	0.57
Serum ascorbic acid, mean (SD)	35.7 (21.1)	38.8 (16.6)	3.1 (−10.3, 16.5)	0.64
Self-reported food insecurity, n (%)				
Yes (score ≥ 2)	16 (80)	14 (70)	-	
No (score < 2)	4 (20)	6 (30)	-	0.72
Self-reported financial insecurity, *n* (%)				
Yes	14 (70)	15 (75)	-	
No	5 (25)	5 (25)	-	
Decline to respond	1 (5)	0 (0)	-	0.60
Self-reported general health, *n* (%)				
Good or very good	14 (70)	12 (60)	-	
Moderate, bad, or very bad	6 (30)	8 (40)		0.51

## Data Availability

The raw data supporting the conclusions of this article will be made available by the authors on request.

## Supplementary Materials

The following supporting information can be downloaded at https://www.mdpi.com/article/10.3390/nu17172865/s1, Table S1: Distribution of daily intake of food group equivalents (in Canada’s Food Guide servings) and nutrients according to trial groups and sex, as reported in the Canadian Diet History Questionnaire (CDHQ) III [27,28]; Table S2: Mean component scores on the Healthy Eating Food Index (HEFI)-2019 according to trial group and sex, as reported in the Canadian Diet History Questionnaire (CDHQ) III.

## Abbreviations

The following abbreviations are used in this manuscript:

HEFI	Healthy Eating Food Index
CDHQ	Canadian Diet History Questionnaire
CFG	Canadian Food Guidelines
HbA1c	Hemoglobin A1c
RA	Recommended allowance
