# Supplementary material for: Assessing the Effect of a Food Voucher on the Dietary Intake of Patients with Diabetes Using the Canadian Diet History Questionnaire III: A Randomized Control Trial
Source: Nutrients. 2025 Sep 4;17(17):2865. doi: 10.3390/nu17172865 (PMC12430127; doi:10.3390/nu17172865)
Supplement: Supplementary file 1 [file nutrients-17-02865-s001.zip › nutrients-3836071-supplementary.pdf]

Assessing the Effect of a Food Voucher on the Dietary Intake of Patients with Diabetes using the Canadian Diet History Questionnaire III: A Randomized Control Trial

Supplementary Materials

Table S1. Distribution of daily intake of food group equivalents (in Canada's Food Guide servings)[1] and Nutrients according to Trial groups and sex, as reported in the Canadian Diet History Questionnaire (CDHQ) III.

	Control		Intervention			
**Male**	**Mean (SD)**	**Min, Max**	**Mean (SD)**	**Min, Max**	**Mean differences (95% CI)***	***p*-Values***
**CFG equivalent in servings[1]**						
Total Fruit and vegetables[2,3]	6.0 (6.3)	1.0, 20.5	6.4 (5.3)	1.5, 15.3	0.4 (−5.8, 6.6)	0.90
Whole Fruits[4]	0.9 (0.9)	0.2, 2.8	2.3 (1.8)	0.6, 5.4	1.4 (0.0, 2.8)	0.05
Dark green vegetables[2]	0.7 (0.7)	0.0, 1.8	0.7 (0.9)	0.0, 2.5	−0.1 (−0.9, 0.8)	0.87
Orange vegetables[2]	0.3 (0.3)	0.0, 1.0	0.3 (0.4)	0.0, 1.1	−0.0 (−0.4, 0.4)	0.90
White potato vegetables[5]	0.2 (0.3)	0.0, 1.0	0.2 (0.3)	0.0, 0.8	−0.1 (−0.4, 0.2)	0.71
Total grains[2]	3.5 (2.3)	0.7, 7.8	1.9 (1.1)	0.3, 3.5	−1.7 (−3.7, 0.3)	0.09
Whole grains[6]	0.5 (0.7)	0.0, 2.1	0.4 (0.5)	0.0, 1.3	−0.1 (−0.7, 0.6)	0.82
Refined grains[7]	2.3 (1.9)	0.7, 7.1	0.7 (0.4)	0.2, 1.3	−1.6 (-3.1, -0.1)	0.04
Milk and alternatives[2,8]	1.3 (1.2)	0.2, 3.9	0.7 (0.5)	0.2, 1.3	−0.6 (−1.6, 0.4)	0.25
Meat and alternatives[2,9]	3.3 (2.1)	0.7, 6.7	2.0 (1.8)	0.3, 4.6	−1.2 (−3.3, 0.8)	0.22
**Other Foods and Nutrients**						
Ultra-processed foods in grams[10]	98.5 (98.2)	21.0, 323.0	68.2 (68.2)	18.1, 217.9	−30.3 (−122.2, 61.6)	0.49
Amount of food in grams	3125.4 (1680.3)	684.6, 6903.2	2867.9 (2248.3)	651.2, 6766.6	−257.6 (−2282.4, 1767.3)	0.79
Energy in kcal	1404.0 (866.3)	380.4, 2753.8	1019.0 (641.3)	246.0, 1837.4	−385.0 (−1208.6, 438.6)	0.33
Total Fat in grams	55.3 (39.1)	13.2, 134.6	36.9 (27.8)	7.0, 84.8	−18.4 (−55.2, 18.4)	0.30
Total Fat in % kcal	34.7 (7.0)	23.8, 45.7	30.9 (7.3)	20.7, 41.6	−3.8 (−11.3, 3.7)	0.29
Saturated Fat in grams	17.4 (11.7)	4.7, 37.4	12.2 (10.7)	2.2, 34.2	−5.2 (−17.1, 6.6)	0.36

Saturated Fat in % kcal	11.0 (2.0)	8.4, 14.4	10.3 (4.1)	5.8, 16.7	−0.7 (−3.9, 2.5)	0.64
Unsaturated Fat in grams	37.9 (27.7)	8.5, 97.1	24.7 (17.8)	4.8, 50.7	−13.2 (−38.6, 12.3)	0.29
Unsaturated fat in % kcal	23.7 (5.3)	15.4, 31.7	20.5 (3.8)	14.9, 24.8	−3.1 (−8.1, 1.9)	0.20
Protein in grams	68.2 (41.1)	17.3, 123.2	44.9 (35.8)	9.5, 99.6	−23.3 (−64.4, 17.7)	0.24
Protein in % kcal	19.7 (5.1)	16.3, 33.6	16.0 (4.4)	10.8, 21.7	−3.7 (−8.7, 1.4)	0.14
Carbohydrates in grams	162.2 (112.2)	47.3, 385.5	133.6 (78.3)	38.4, 264.3	−28.5 (−133.6, 76.5)	0.57
Carbohydrates in % kcal	46.3 (9.5)	31.1, 62.8	56.1 (12.5)	37.4, 73.8	9.8 (−1.6, 21.1)	0.09
Sodium in mg	2545.9 (1916.1)	674.7, 6474.5	1762.3 (1575.6)	413.1, 4696.8	−783.6 (−2661.3, 1094.2)	0.39
**Female**						
**CFG equivalent in servings**[1]						
Total Fruit and vegetables[2,3]	6.4 (5.2)	1.8, 15.7	6.4 (3.2)	2.5, 12.1	0.0 (−3.7, 3.6)	0.99
Whole Fruits[4]	1.8 (1.0)	0.2, 3.8	2.1 (1.1)	0.5, 4.4	0.3 (−0.6, 1.3)	0.47
Dark green vegetables[2]	1.4 (2.2)	0.0, 6.6	1.3 (1.0)	0.1, 2.9	−0.1 (−1.5, 1.3)	0.87
Orange vegetables[2]	0.4 (0.6)	0.0, 1.9	0.3 (0.3)	0.0, 1.2	−0.1 (−0.5, 0.3)	0.66
White potato vegetables[5]	0.3 (0.5)	0.0, 1.8	0.3 (0.2)	0.3, 0.8	0.0 (−0.4, 0.3)	0.89
Total grains[2]	2.9 (1.8)	1.0, 6.1	3.0 (2.2)	0.5, 7.2	0.2 (−1.6, 1.9)	0.86
Whole grains[6]	0.3 (0.4)	0.0, 1.2	0.8 (1.0)	0.0, 3.5	0.5 (−0.2, 1.2)	0.13
Refined grains[7]	2.1 (1.9)	0.4, 6.0	1.5 (1.1)	0.3, 3.6	−0.6 (−1.9, 0.7)	0.35
Milk and alternatives[2,8]	1.3 (1.0)	0.2, 3.4	0.9 (0.6)	0.1, 2.2	−0.4 (−1.0, 0.3)	0.30
Meat and alternatives[2,9]	2.0 (1.7)	0.5, 6.4	2.2 (1.4)	0.4, 4.7	0.2 (−1.1, 1.5)	0.79
**Other foods and Nutrients**						
Ultra-processed foods in grams[10]	48.0 (33.6)	14.5, 120.7	55.0 (41.9)	13.2, 143.8	6.9 (−26.8, 40.7)	0.67
Amount of food in grams	3152.1 (3417.2)	681.2, 12696.1	4155.4 (2986.0)	1961.8, 11349.8	1003.2 (−1776.6, 3783.1)	0.46
Energy in kcal	1210.5 (531.5)	568.8, 2488.8	1242.4 (440.1)	538.6, 1916.5	31.9 (−389.2, 453.0)	0.88
Total Fat in grams	41.9 (23.0)	20.3, 97.1	47.4 (22.3)	14.4, 84.5	5.5 (−14.2, 25.3)	0.57

Total Fat in % kcal	30.7 (6.4)	18.5, 38.0	33.4 (8.1)	14.4, 47.3	2.7 (−3.8, 9.2)	0.39
Saturated Fat in grams	12.7 (5.8)	5.3, 22.5	15.2 (8.9)	2.9, 28.4	2.6 (−4.2, 9.3)	0.44
Saturated Fat (% kcal)	9.5 (2.7)	6.0, 14.2	10.5 (3.8)	2.9, 15.5	1.0 (−1.9, 4.0)	0.48
Unsaturated Fat in grams	29.3 (18.1)	14.5, 74.6	32.2 (14.0)	11.1, 57.5	3.0 (−10.9, 16.8)	0.66
Unsaturated fat in % kcal	21.2 (5.1)	10.9, 27.6	22.9 (5.2)	11.5, 33.8	1.7 (−2.9, 6.2)	0.45
Protein in grams	47.1 (26.5)	28.6, 117.6	50.8 (23.4)	16.6, 83.2	3.7 (−18.0, 25.4)	0.73
Protein in % kcal	15.6 (3.1)	10.7, 20.1	16.1 (3.9)	7.4, 20.2	0.5 (−2.6, 3.6)	0.73
Carbohydrates in grams	169.4 (69.1)	71.2, 313.5	158.1 (46.8)	76.9, 232.6	−11.2 (−61.5, 39.0)	0.65
Carbohydrates in % kcal	56.4 (8.0)	46.0, 69.4	52.6 (11.2)	40.6, 83.5	−3.7 (−12.5, 5.0)	0.39
Sodium in mg	1711.6 (929.7)	871.8, 3919.4	1878.4 (794.4)	763.5, 3177.6	166.9 (−581.0, 914.8)	0.65

[1]Servings according to "Eating Well with Canada's Food Guide". Sourced from Health Canada. Canada's Food Guide, 2007. Available online: https://publications.gc.ca/collections/Collection/H164-38-1-2007E.pdf (accessed on 3 June 2025) [27].

[2]Foods acceptable in the food group according to CFG-2007.

[3]Includes 100% fruit and vegetable juice.

[4]Includes all forms except juice.

[5]Regardless of cooking method (e.g., fries).

[6]All grain foods reported as whole grain in CDHQ III.

[7]All grain foods not reported as whole grain in CDHQ III.

[8]Includes all milk products, such as fluid milk, yogurt, cheese, and fortified soy beverages.

[9]Includes seafood, nuts, seeds, soy products (other than beverages), and legumes (beans and peas).

[10]Ultra-processed foods as defined by Monteiro, C.A.; Cannon, G.; Levy, R.B.; Moubarac, J.-C.; Louzada, M.L.; Rauber, F.; Khandpur, N.; Cediel, G.; Neri, D.; Martinez-Steele, E.; et al. Ultra-Processed Foods: What They Are and How to Identify Them. *Public Health Nutr* 2019, *22*, 936–941, doi:10.1017/S1368980018003762 [28].

[*]Statistically significant values were evaluated using a two-sample t-test at $p<0.05$ and a 95% confidence interval not enclosing "0" (value representing "no effect") for between-group differences in means ($n=40$).

Table S2. Mean component scores on the Healthy Eating Food Index (HEFI)-2019 according to trial group and sex, as reported in the Canadian Diet History Questionnaire (CDHQ) III.

	Control		Intervention			
**Components (Maximum point possible)**	**Mean (SD)**	**Min, Max**	**Mean (SD)**	**Min, Max**	**Mean differences (95% CI)***	***p*-Values***
**Male**						
**Food & Beverage Intake**						
1 Vegetables and Fruits (20)	15.1 (3.7)	8.2, 20.0	19.2 (1.4)	16.8, 20.0	4.1 (0.9, 7.2)	0.02
2 Whole-grain foods (5)	0.9 (1.1)	0.0, 3.1	0.7 (0.9)	0.0, 2.5	−0.2 (−1.3, 0.9)	0.67
3 Grain foods ratio (5)	0.9 (0.9)	0.0, 2.6	1.1 (1.1)	0.0, 3.2	0.2 (−0.8, 1.2)	0.68
4 Protein foods (5)	5.0 (0.0)	5.0, 5.0	3.9 (1.5)	1.4, 5.0	−1.1 (−2.1, −0.1)	0.03
5 Plant-based protein foods (5)	1.0 (1.6)	0.0, 4.8	0.6 (0.8)	0.0, 2.2	−0.4 (−1.8, 1.0)	0.58
6 Beverages (10)	8.7 (1.3)	6.6, 9.9	6.9 (3.2)	1.3, 9.7	−1.8 (−4.2, 0.6)	0.13
Subgroup 1 Total Score	31.6 (3.8)	27.1, 37.4	32.3 (4.6)	25.4, 38.0	0.8 (−3.6, 5.1)	0.71
**Nutrient Intake**						
7 Fatty acids ratio (5)	2.4 (0.8)	1.4, 4.1	2.5 (1.8)	0.4, 5.0	0.1 (−1.3, 1.5)	0.85
8 Saturated fats (5)	3.6 (1.5)	0.6, 5.0	3.4 (2.2)	0.0, 5.0	−0.2 (−2.1, 1.7)	0.81
9 Free sugars (10)	9.6 (1.2)	6.2, 10.0	6.7 (3.4)	0.0, 10.0	−2.9 (−5.4, −0.5)	0.02
10 Sodium (10)	3.0 (2.3)	0.0, 5.9	4.6 (3.6)	0.0, 8.9	1.6 (−1.5, 4.6)	0.28
Subgroup 2 Total Score	18.7 (3.8)	12.6, 23.2	17.2 (3.8)	10.4, 21.5	−1.5 (−5.4, 2.5)	0.45
HEFI Total Score (80)	50.2 (6.6)	41.8, 59.4	49.5 (7.5)	35.9, 59.5	−0.7 (−8.0, 6.6)	0.84
**Female**						
**Food & Beverage Intake**						
1 Vegetables and Fruits (20)	17.2 (4.5)	5.9, 20.0	17.7 (3.9)	8.3, 20.0	−0.5 (−3.2, 4.1)	0.80
2 Whole-grain foods (5)	0.7 (0.9)	0.1, 2.5	1.0 (0.9)	0.0, 2.6	0.3 (−0.5, 1.1)	0.42
3 Grain foods ratio (5)	1.2 (1.5)	0.1, 4.0	1.4 (1.3)	0.0, 3.9	0.3 (−0.9, 1.5)	0.65
4 Protein foods (5)	4.1 (1.5)	1.1, 5.0	4.4 (1.0)	2.3, 5.0	0.3 (−0.8, 1.4)	0.59
5 Plant-based protein foods (5)	0.8 (0.8)	0.0, 2.5	1.4 (1.7)	0.1, 5.0	0.6 (−0.6, 1.9)	0.31

6 Beverages (10)	8.0 (2.2)	2.9, 10.0	9.2 (1.5)	4.2, 10.0	1.2 (−0.4, 2.8)	0.13
Subgroup 1 Total Score	31.9 (7.3)	19.4, 41.6	35.1 (4.6)	24.6, 41.0	3.2 (−2.0, 8.3)	0.22
**Nutrient Intake**						
7 Fatty acids ratio (5)	2.9 (1.9)	0.2, 5.0	3.1 (1.8)	0.2, 5.0	0.2 (−1.4, 1.8)	0.76
8 Saturated fats (5)	4.2 (1.8)	0.8, 5.0	3.2 (2.1)	0.0, 5.0	−1.0 (−2.7, 0.7)	0.23
9 Free sugars (10)	7.5 (2.6)	1.8, 10.0	8.1 (3.2)	0.0, 10.0	0.6 (−1.9, 3.2)	0.61
10 Sodium (10)	5.4 (2.9)	0.4, 10.0	4.5 (3.2)	0.0, 9.9	−0.9 (−3.6, 1.8)	0.49
Subgroup 2 Total Score	19.9 (4.1)	13.0, 24.8	18.9 (3.4)	13.4, 27.4	−1.0 (−4.3, 2.2)	0.51
HEFI Total Score (80)	51.8 (9.7)	35.0, 65.1	53.9 (5.9)	45.8, 65.5	2.1 (−4.7, 8.9)	0.52

*Statistically significant values were evaluated using a two-sample t-test at $p<0.05$ and a 95% confidence interval not enclosing "0" (value representing "no effect") for between-group differences in means (n=40).
